# EHMTI-0030. Migraine pain location in adult: the bangladeshi experience

**DOI:** 10.1186/1129-2377-15-S1-D27

**Published:** 2014-09-18

**Authors:** R Haque

**Affiliations:** 1 Neuro Medicine, Uttara Adhunik Medical College, Dhaka, Bangladesh

## Background

Sparse literature documenting the location of pain at the onset of migraine attacks and during established headaches is available especially of Bangladesh.

## Objectives

A prospective study (2011–12) on 500 adult migraine patients (International Classifications of Headache Disorders (ICHD), 2:1.1, 1.2.1 and 1.6.1) was conducted to document (a) sites of onset of pain and (b) location of pain during established attacks (in >50% occasions) through semi structured interviews.

## Results

### Demography

N = 500; M:F = 136:364 (1:2.68); age, 15–50 years (mean, 26 years); duration of migraine, 1–20 years (mean, 6.8 years). 100% of the subjects were Bangladeshi, Dhaka being the main city.

### Migraine types

(On the basis of >50% headache spells): N = 500; 1.1:420 (84.0%); 1.2.1:13 (2.6%); 1.6.1:75(15.0%).

### Location of pain at onset

Unilateral onset was present in 42.2% of the patients; of these, 54.0% had eye pain; 9.0%, frontal pain and 35.3%, temporal pain. In 36.2% of the patients, bilateral/central location of pain, mostly bitemporal or at vertex was noted. Cervico-occipital pain onset was noted in 21.6% patients (of them predominantly occipital, 61.2%; predominantly cervical, 38.8%).

### Location of established headaches

In 47.0% of the patients, with unilateral ocular or temporal onset, pain remained at the same site. Pain became hemicranial in 38.4%. In most patients, unilateral frontal onset pain (56.5%) became bilateral or holocranial. Most bilateral ocular (70.2%) and temporal onset (68.4%) pains remained at the same location. However, most bifrontal (57.8%) and vertex onset (57.8%) pains subsequently became holocranial. Most occipital pains at onset became holocranial (46.3%), but cervical pains subsequently became either hemicranial (40.2%) or holocranial (35.2%).

## Conclusions

This study documents location of pain at the onset and during established headaches in migraine patients largely from Dhaka, Bangladesh. Migraine with aura appears to be rare among Bangladeshi. More than half had onset pain bilaterally/centrally and in the cervico-occipital regions. Only 40.5% experienced only unilateral pain. Cervico-occipital migraine pain appears to be common in Bangladeshi.

No conflict of interest.

